# First person – Andrew Scott Emmert and Eri Iwasawa

**DOI:** 10.1242/dmm.043026

**Published:** 2019-11-21

## Abstract

First Person is a series of interviews with the first authors of a selection of papers published in Disease Models & Mechanisms (DMM), helping early-career researchers promote themselves alongside their papers. Andrew Scott Emmert and Eri Iwasawa are co-first authors on ‘ Impaired neural differentiation and glymphatic CSF flow in the *Ccdc39* rat model of neonatal hydrocephalus: genetic interaction with *L1cam*’, published in DMM. Andrew Scott is a medical student in the lab of Dr June Goto and Dr Francesco T. Mangano at Cincinnati Children's Hospital Medical Center, investigating the generation and characterization of transgenic rodent models of pediatric hydrocephalus. Eri is a post-doctoral fellow in the same lab, investigating the pathogenesis of hydrocephalus in order to develop new therapeutics for the disease.


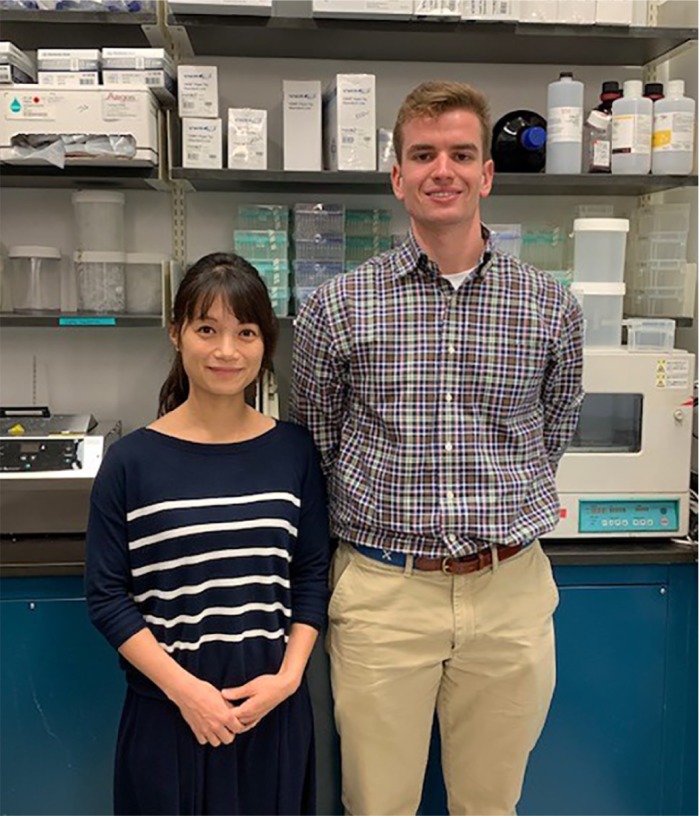


**Eri Iwasawa and Andrew Scott Emmert**

**How would you explain the main findings of your paper to non-scientific family and friends?**

ASE: Pediatric hydrocephalus, or ‘water on the brain’, is a life-threatening condition that affects about 1 in every 1000 children. Although this condition can be treated with brain surgery, no cure exists for children living with the debilitating features of this disease. In our current paper, we used a cutting-edge technology, CRISPR/Cas9 genome editing, to develop a rat model of a hydrocephalus-causing gene mutation, *Ccdc39^prh^*, that our lab previously found in mice. Rats offer a number of advantages over mice for studying hydrocephalus in the laboratory, mainly due to their large anatomical size for imaging and surgical experiments. We found that rats with this mutation gradually develop massive hydrocephalus within 2 weeks after birth and most of them die within a month. When our new hydrocephalus gene was combined with another model of X-linked hydrocephalus, the resulting ‘double-mutant’ rats were dramatically sicker with worse hydrocephalus than the single-mutant rats. Another part of our study focused on a recently discovered system of fluid clearance in the brain, the glymphatic system, which is slowed in conditions such as Alzheimer's disease and traumatic brain injury. We discovered that this system is also impaired in our neonatal hydrocephalus model, which suggests that the glymphatic system may be a good therapeutic target for developing non-surgical treatments for hydrocephalus.

“Rats offer a number of advantages over mice for studying hydrocephalus in the laboratory, mainly due to their large anatomical size for imaging and surgical experiments.” *– Andrew Scott Emmert*

EI: Cerebral spinal fluid (CSF) is a transparent fluid inside and around the brain that keeps the brain healthy and plays important roles, more than a shock absorber. The CSF is continuously produced by the choroid plexus cells of the brain and absorbed back into the body. Hydrocephalus, a very common brain anomaly especially in premature babies, is a condition that appears when this circulation is distracted by excessive production of CSF, or obstruction within the CSF pathway/absorption. The accumulation of CSF within the brain ventricles leads to brain damage and eventually death if left untreated. In this study, we developed a rat model of congenital hydrocephalus that shows neonatal hydrocephalus due to the malfunction of ependymal cilia-mediated CSF circulation. This model shows a delay in brain development, similar to the human neonatal hydrocephalus patients. Interestingly, inflammatory reaction in the brain and subarachnoid space was observed without any infection. We think that this inflammatory reaction is involved in the pathogenesis of hydrocephalus and may accelerate the severity of the disease, and therefore is worth investigating further to find anti-inflammatory drugs to treat hydrocephalus.

**What are the potential implications of these results for your field of research?**

ASE: Our paper demonstrates the influences of two genes on the development of hydrocephalus and that specific mutations in both of these genes may ‘talk’, or epistatically interact, with one another to cause a more severe form of the disease as compared to when either of these genes is mutated individually. This ‘gene talking’ could explain why some children with hydrocephalus are sicker than others. Our finding of an important pro-inflammatory signal in the ‘attack’ on the brain that results from hydrocephalus provides a new opportunity for treating hydrocephalus with drugs. In addition to providing a new therapeutic target, the glymphatic system impairment in this model may explain why pediatric hydrocephalus patients experience cognitive delays. Overall, this new model provides greater flexibility in the type of experiments, such as CSF diversion surgery, that can be used to study how brain development is affected by pediatric hydrocephalus.

**Figure:**
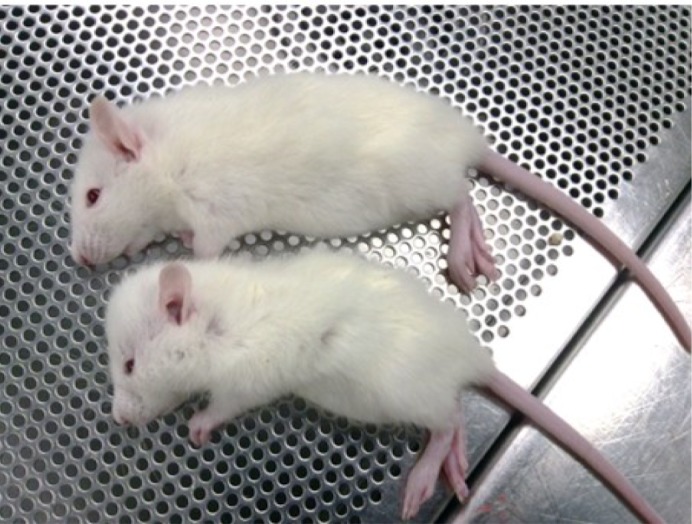
**Gross picture of normal (top) and hydrocephalic (bottom) rats shows the dome-shaped head of a mutant rat at postnatal day 25.**

**What are the main advantages and drawbacks of the model system you have used as it relates to the disease you are investigating?**

EI: The primary advantage of this model is that we used rats instead of mice, which enables us to surgically intervene to reduce CSF in future studies. We can then model the surgically treated pediatric brain in animals. More forms of behavioral testing are available for rats than mice, and we can use these tests to evaluate treatment outcomes in terms of cognitive and motor skill functions. This characteristic is valuable to assess the long-term effect of the drug in the future. The drawback of the rat model is the potential difference in their CSF outflow pathways compared to those of humans. The major routes of CSF outflow in the human brain, arachnoid villi, develop later in life in the mature human brain but not in the rodent brain.

**What has surprised you the most while conducting your research?**

ASE: I have been most pleasantly surprised by the tremendous community of hydrocephalus researchers, patients and families who collaborate to improve the understanding and treatment of hydrocephalus. Even though hydrocephalus is a relatively rare condition, this disease dramatically affects the lives of the patients and families living with it. Yet, my experience meeting providers, scientists and patients at national conferences like the Society for Research into Hydrocephalus and Spina Bifida has shown me that many patients and families are eager to embrace biomedical research as a way to improve the treatment of this condition for future patients. Such collaboration and support are invigorating for an aspiring physician-scientist like myself.

EI: The accumulation of inflammatory cells in the subarachnoid space of our hydrocephalus model surprised me most since the lateral ventricle expansion was thought to be the main cause of insult in hydrocephalus. This finding encouraged me to fully understand the physiology, especially concerning CSF circulation.

**Describe what you think is the most significant challenge impacting your research at this time and how will this be addressed over the next 10 years?**

ASE: One of the greatest challenges impacting pediatric hydrocephalus research is the availability of experimental tools to investigate the genetic basis of this condition. While not all forms of hydrocephalus are due to genetic causes, nearly 40% of neonatal hydrocephalus cases are due to abnormal genetics. Yet, new technology to understand the genetic basis of hydrocephalus has only recently become available. Cutting-edge technologies like CRISPR/Cas9 gene editing and whole-exome sequencing have led to the identification of new gene mutations and hydrocephalus models that have improved our understanding of this condition. I expect that these technologies and others that become available in the next 10 years will greatly improve our understanding of hydrocephalus.

EI: The most significant challenge in developing new therapeutics for pediatric hydrocephalus is that pediatric hydrocephalus contains multiple etiologies, including congenital hydrocephalus (known and unknown genetic mutations) and acquired hydrocephalus (intraventricular hemorrhage, infection, neoplasms). Even with the same gene mutation, the phenotypes sometimes differ. Our model results in 100% penetrant hydrocephalus without any drugs/blood, which enables us to study the phenomena following the ventriculomegaly. Still, this variability in etiology can be an obstacle to drug treatment for humans. In the future, different therapeutics will be used depending on the etiology or particular gene mutation causing the hydrocephalus.

“In the future, different therapeutics will be used depending on the etiology or particular gene mutation causing the hydrocephalus.” *– Eri Iwasawa*

**What changes do you think could improve the professional lives of early-career scientists?**

EI: More opportunities to discuss frankly with people from many different backgrounds, more grants for young investigators, and early exposure to other experiences (lab work itself, presentation and mentoring) might improve the professional lives of early-career scientists.

ASE: To encourage medical students or residents to incorporate hydrocephalus research into their careers, greater integration of research training during medical school or clinical residency is essential. As hydrocephalus is a relatively uncommon condition, early promotion of the need for research in the field is essential as students or residents select a particular academic focus.

**What's next for you?**

ASE: I am a first-year medical student at the University of Cincinnati College of Medicine. I aspire for a career in academic medicine and research, and my experiences in the lab have fostered my interest in caring for pediatric patients with neurological conditions.

EI: I am a medical doctor with a neurology background, and am currently focusing on basic research to find better therapeutic options for neurological diseases. I will further investigate hydrocephalus pathophysiology and its possible treatment.
